# Supplementary material for: Individual differences in spatial working memory strategies differentially reflected in the engagement of control and default brain networks
Source: Cereb Cortex. 2024 Aug 30;34(8):bhae350. doi: 10.1093/cercor/bhae350 (PMC11364466; doi:10.1093/cercor/bhae350)
Supplement: SupplementaryMaterial_bhae350 [file supplementarymaterial_bhae350.pdf]

Supplementary material: Individual differences in spatial working memory strategies differentially reflected in the engagement of control and default brain networks

Nina Purg Suljič[a*], Aleksij Kraljič[a], Masih Rahmati[b], Youngsun T. Cho[b], Anka Slana Ozimič[a], John D. Murray[b,c,d], Alan Anticevic[b,c+], and Grega Repovš[a+]

[a]Department of Psychology, Faculty of Arts, University of Ljubljana, Aškerčeva 2, 1000 Ljubljana, Slovenia
[b]Department of Psychiatry, Yale University School of Medicine, 300 George Street, New Haven, CT 06511, USA
[c]Department of Psychology, Yale University, 100 College Street, New Haven, CT 06510, USA
[d]Department of Physics, Yale University, 217 Prospect Street, New Haven, CT 06511, USA
[*]Corresponding author
[+]Co-senior authors

Supplementary tables

Table S1. Demographic data of participants included in the data analysis

Study	Number		Age (years)				Handedness			Education (years)		
	All	Females	Range	All (SD)	Females (SD)	Males (SD)	Right	Left	Both	All (SD)	Females (SD)	Males (SD)
**I**	27	18	18–38[2]	23.3 (5.8)[2]	20.9 (3.0)[2]	27.6 (7.2)	25	2	0	14.5 (2.3)[4]	13.9 (2.2)[3]	15.8 (2.1)[1]
**II**	26	17	19–31	23.0 (3.0)	23.1 (3.5)	22.9 (2.0)	24	0	2	15.4 (2.2)[2]	15.3 (2.6)[2]	15.6 (1.7)
**III**	30	22	19–42	24.5 (5.6)	22.9 (4.7)	28.8 (5.7)	29	0	1	15.1 (2.7)	14.5 (2.7)	16.5 (2.1)
**IV**	37	9	21–36	25.5 (3.4)	25.6 (3.3)	25.5 (3.5)	32	5	0	16.7 (1.7)	17.1 (1.6)	16.6 (1.7)
**V**	25	10	20–40	27.8 (5.9)	26.7 (5.8)	28.5 (6.0)	21[1]	3[1]	0[1]	16.3 (2.5)	16.5 (2.3)	16.2 (2.7)
**VI**	10	1	17–31	22.7 (4.4)	18.0 (–)	23.2 (4.3)	9	1	0	13.9 (2.0)	12.0 (–)	14.1 (2.0)
**All**	155	77	17–42[2]	24.7 (5.0)[2]	23.3 (4.5)[2]	26.1 (5.1)	140[1]	11[1]	3[1]	15.6 (2.4)[6]	15.1 (2.6)[5]	16.0 (2.1)[1]

[1] Missing information for 1 participant.
[2] Missing information for 2 participants.
[3] Missing information for 3 participants.
[4] Missing information for 4 participants.
[5] Missing information for 5 participants.
[6] Missing information for 6 participants.

Table S2. Task parameters used in different studies

Study		I	II	III	IV	V	VI
**Task**	Trials	36	32	24	20	20	80
	Blocks	2	2	3	2	1	4
**Stimuli**	Diameter (px / °va)	100 / 1.06	200 / 2.12	200 / 2.12, 2.83	125 / 1.72	125 / 1.72	125 / 1.72
	Angles (°)	5–355 (steps of 10)	7.5–352.5 (steps of 15)	7.5–352.5 (steps of 15)	9–351 (steps of 18)	9–351 (steps of 18)	9–351 (steps of 18)
	Amplitude (px / °va)	400 / 4.24	400 / 4.24	400 / 4.24, 5.66	415 / 5.72	415, 390 / 5.72, 5.38	415, 390 / 5.72, 5.38
**Events**	Fixation (s)	2.5	2.5	2.5	–	–	–
	Target (s)	0.1	2	2	1.4	1.6	1.6
	Mask (s)	0.05	–	–	–	–	–
	Delay (s)	9.85	8	8	9.8	10.4	10.4
	Attention cue (s)	–	–	–	1.4	1.6	1.6
	Response (s)	3	3	3	2.8	3.2	3.2
	ITI (s)	12.5, 15, 17.5	12.5, 15, 17.5	12.5, 15, 17.5	13.3	15.2	15.2
	ITI ratio	3:2:1	5:2:1	5:2:1	–	–	–
**Screen**	Size (mm)	640 x 400	640 x 400	640 x 400	427 x 343	427 x 343	427 x 343
	Resolution (px)	2560 x 1600	2560 x 1600	2560 x 1600, 1920 x 1200	1280 x 1024	1280 x 1024	1280 x 1024
	Viewing distance (mm)	1350	1350	1350	1385	1385	1385

Table S3. MRI parameters used in different studies

Study		I	II	III	IV	V	VI
**Scanner**		Philips Achieva 3.0T TX			Siemens 3T Tim Trio or Prisma		
**T1w and T2w**	Sagittal slices	236	236	236	224	208	208
	FOV (mm)	224 x 235	224 x 235	224 x 235	256 x 256	256 x 256	256 x 256
	Voxel size (mm)	0.7	0.7	0.7	0.8	0.8	0.8
	TR (ms)	T1: 12, T2: 2500	T1: 12, T2: 2500	T1: 12, T2: 2500	T1: 2400, T2: 3200	T1: 2400, T2: 3200	T1: 2400, T2: 3200
	TE (ms)	T1: 5.7, T2: 414	T1: 5.7, T2: 403	T1: 5.7, T2: 403	T1: 2.07, T2: 564	T1: 2.22, T2: 563	T1: 2.22, T2: 563
	Flip angle (°)	T1: 8, T2: 90	T1: 8, T2: 90	T1: 8, T2: 90	T1: 8, T2: T2 var	T1: 8, T2: T2 var	T1: 8, T2: T2 var
**BOLD**	Axial slices	48	48	48	54	72	72
	FOV (mm)	240 x 240	240 x 240	240 x 240	210 x 210	208 x 208	208 x 208
	Voxel size (mm)	3	3	3	2.5	2	2
	TR (ms)	2500	2500	2500	700	800	800
	TE (ms)	27	27	27	31	37	37
	Flip angle (°)	90	90	90	55	52	52
	SENSE factor 2	2	2	2	–	–	–
	Multi-band factor	–	–	–	6	8	8
	Number of runs	2	2	3	2	1	4
	Frames per run	215	189	281	400	770	770
**Field maps**	Axial slices	48	48	48	54	72	72
	FOV (mm)	240 x 240	240 x 240	240 x 240	210 x 210	208 x 208	208 x 208
	Voxel size (mm)	3	3	3	2.5	2	2
	TR (ms)	2500	2500	2500	731	8000	8000
	TE (ms)	27	27	27	4.92/7.38	66	66
	Flip angle (°)	90	90	90	50	90	90

Supplementary figures

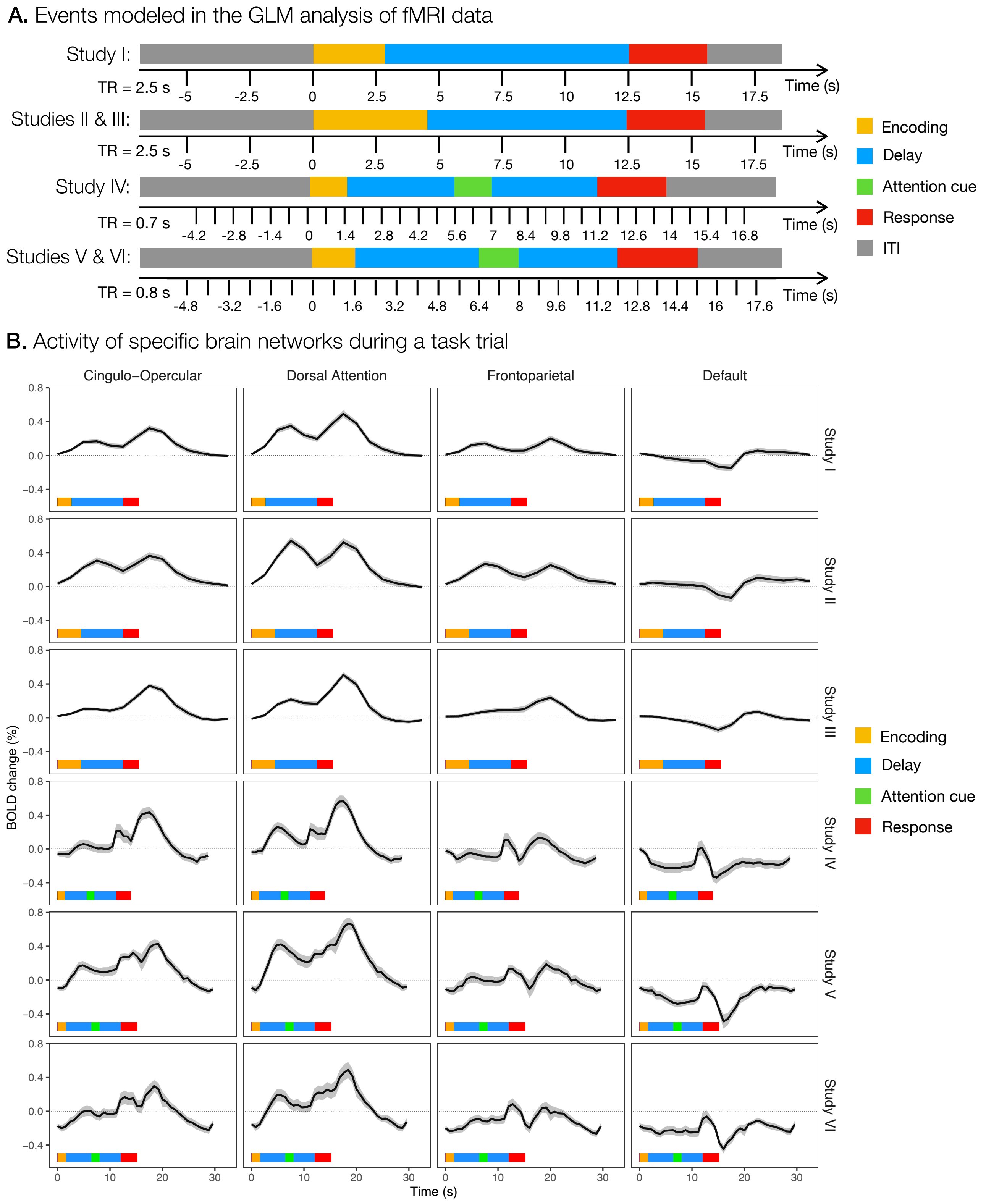

Figure S1. Modeling of the brain activity in specific brain networks during a task trial. A. The timeline of events during a task trial included in the general linear modeling (GLM) in the fMRI data analysis. Zeros mark the start of a task trial. **B.** The average activity is shown for the cingulo-opercular, dorsal attention, frontoparietal, and default networks during a task trial. The shaded area represents the standard error. Colored rectangles mark the timing of different events during a task trial in different studies.

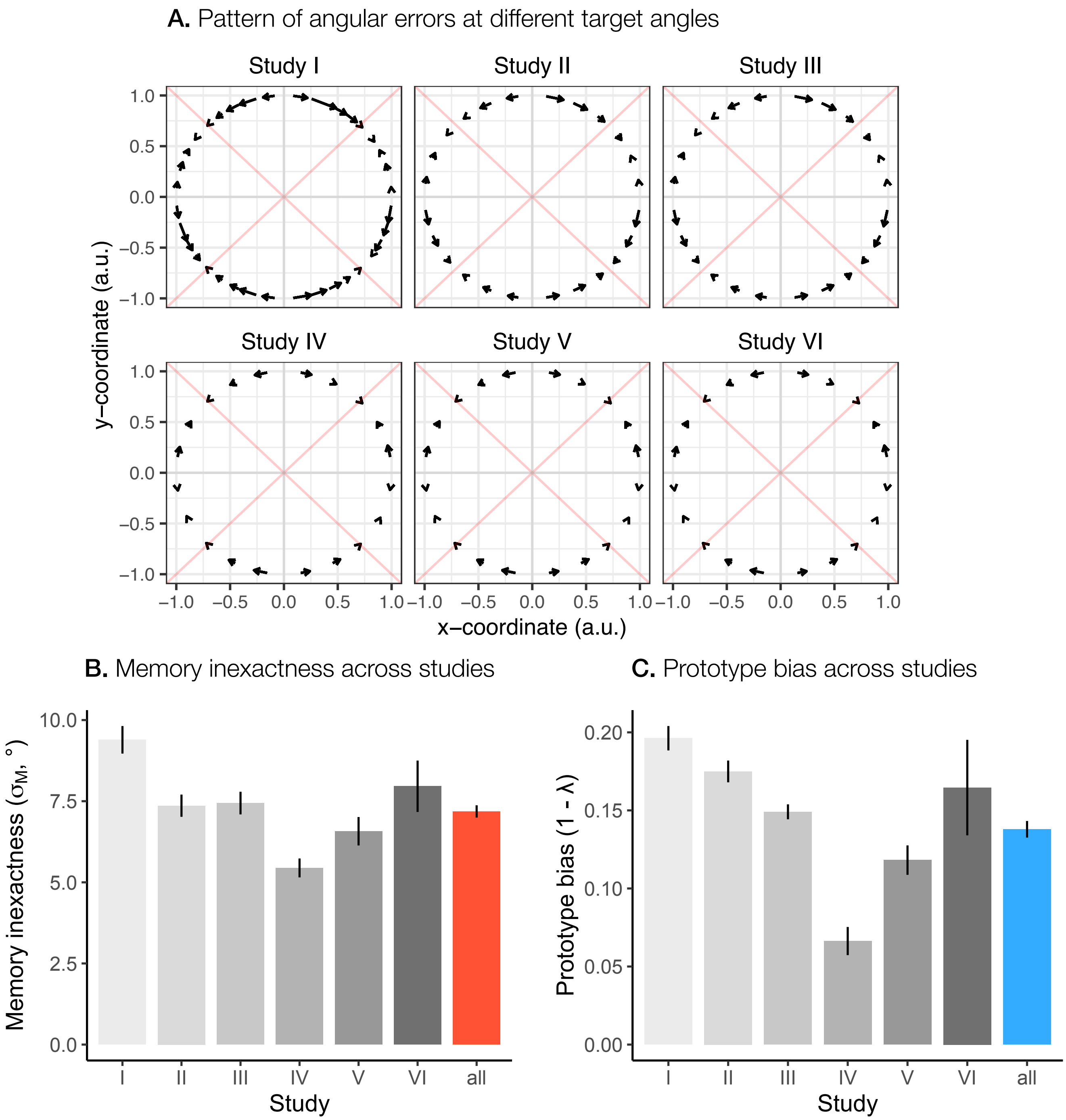

Figure S2. Systematic biases in behavioral performance of the spatial working memory task. A. Pattern of average response errors at different target angles for individual studies. The start of the arrow denotes the target position, while the head of the arrow points to the average response position. **B.** Average memory inexactness across all participants (red) and for individual studies. The error bars represent the standard error. **C.** Average prototype bias across all participants (blue) and for individual studies. The error bars represent the standard error.

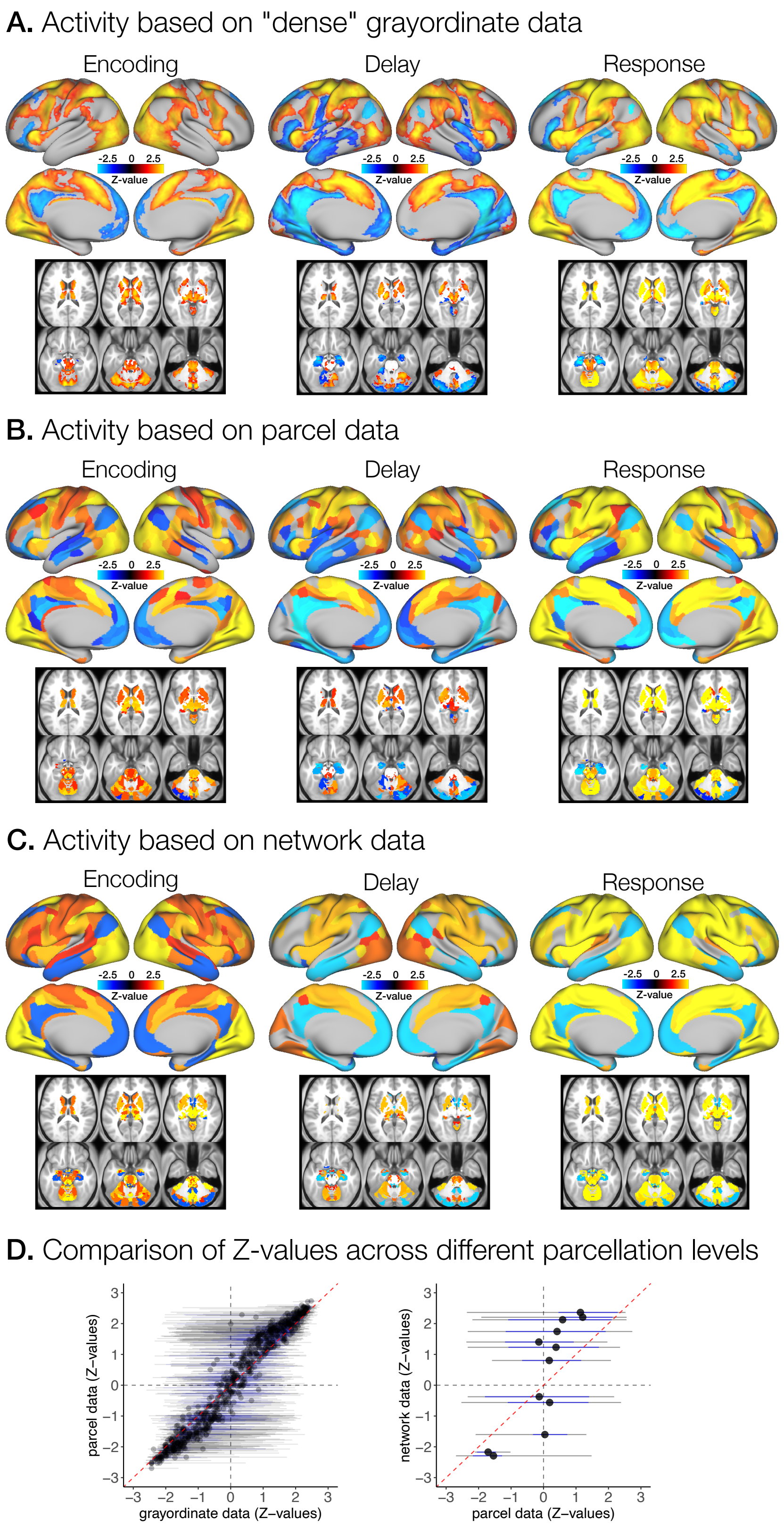

Figure S3. Brain activity during the task based on different levels of fMRI data parcellation. Significant activation and deactivation during the encoding, delay, and response phases for **A.** "dense" grayordinate, **B.** parcel, and **C.** network fMRI data. p-values for "dense" grayordinate data were corrected for multiple comparisons with TFCE FWE, whereas p-values for parcel and network data were corrected with FDR. All images were thresholded at $p < 0.05$. **D.** The comparison of unthresholded Z-value maps for delay-related activity between "dense" grayordinate and parcel data, and additionally, for parcel and network data. The gray lines represent the range, the blue lines the inter-quartile range (IQR), and the red dashed line the diagonal.

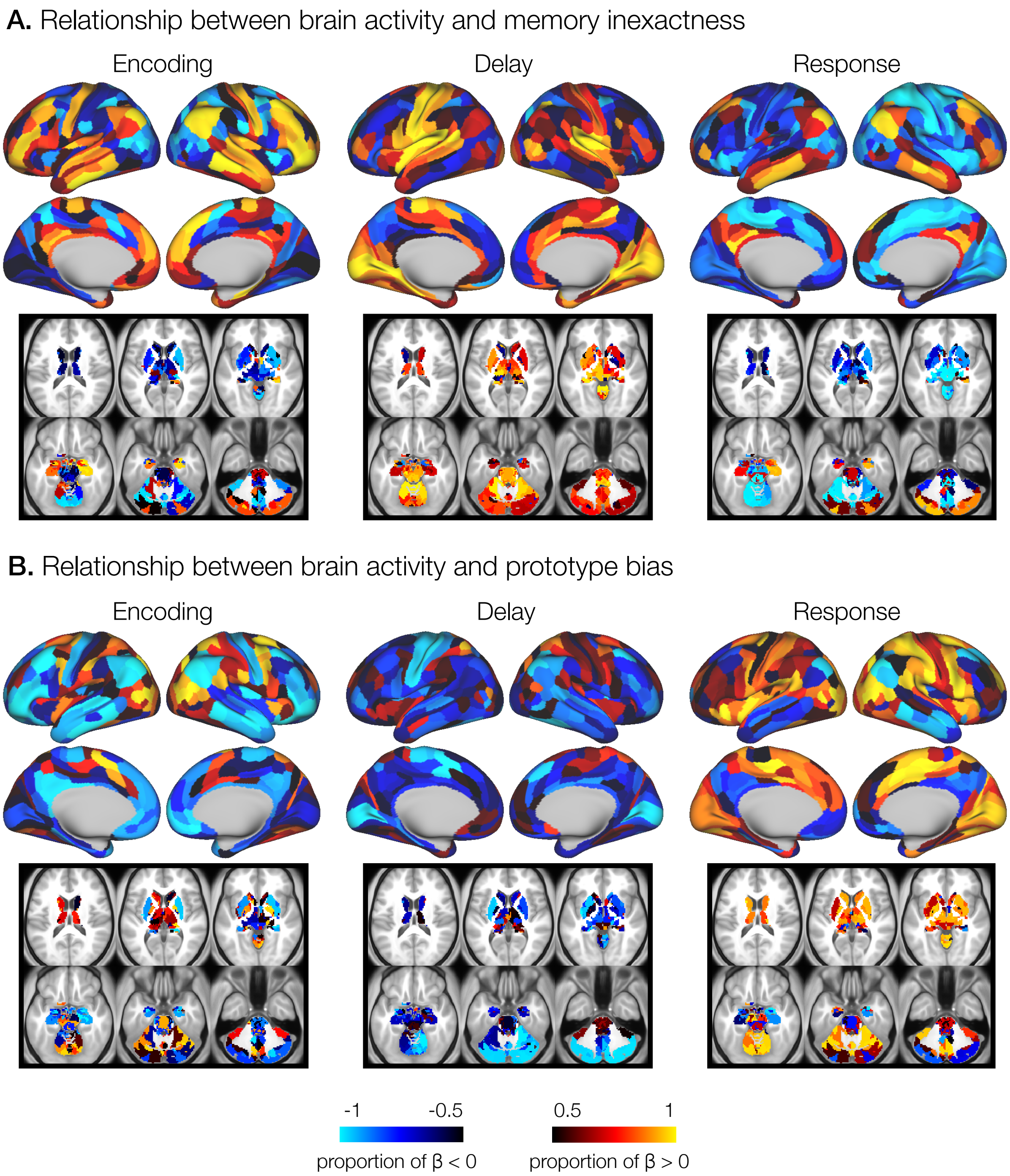

Figure S4. The relationship between the activity of different brain regions and individual spatial working memory performance. The relationship between brain activity and behavioral measures was estimated by running a Bayesian hierarchical linear model across participants with factors memory inexactness and prototype bias, and study as a random effect for each task phase separately. Shown are the proportions of a posterior distribution below or above 0 for the relationship between the activity in each brain area and behavioral measures of **A.** memory inexactness and **B.** prototype bias. The results are presented for the encoding, delay, and response phases of a task trial.

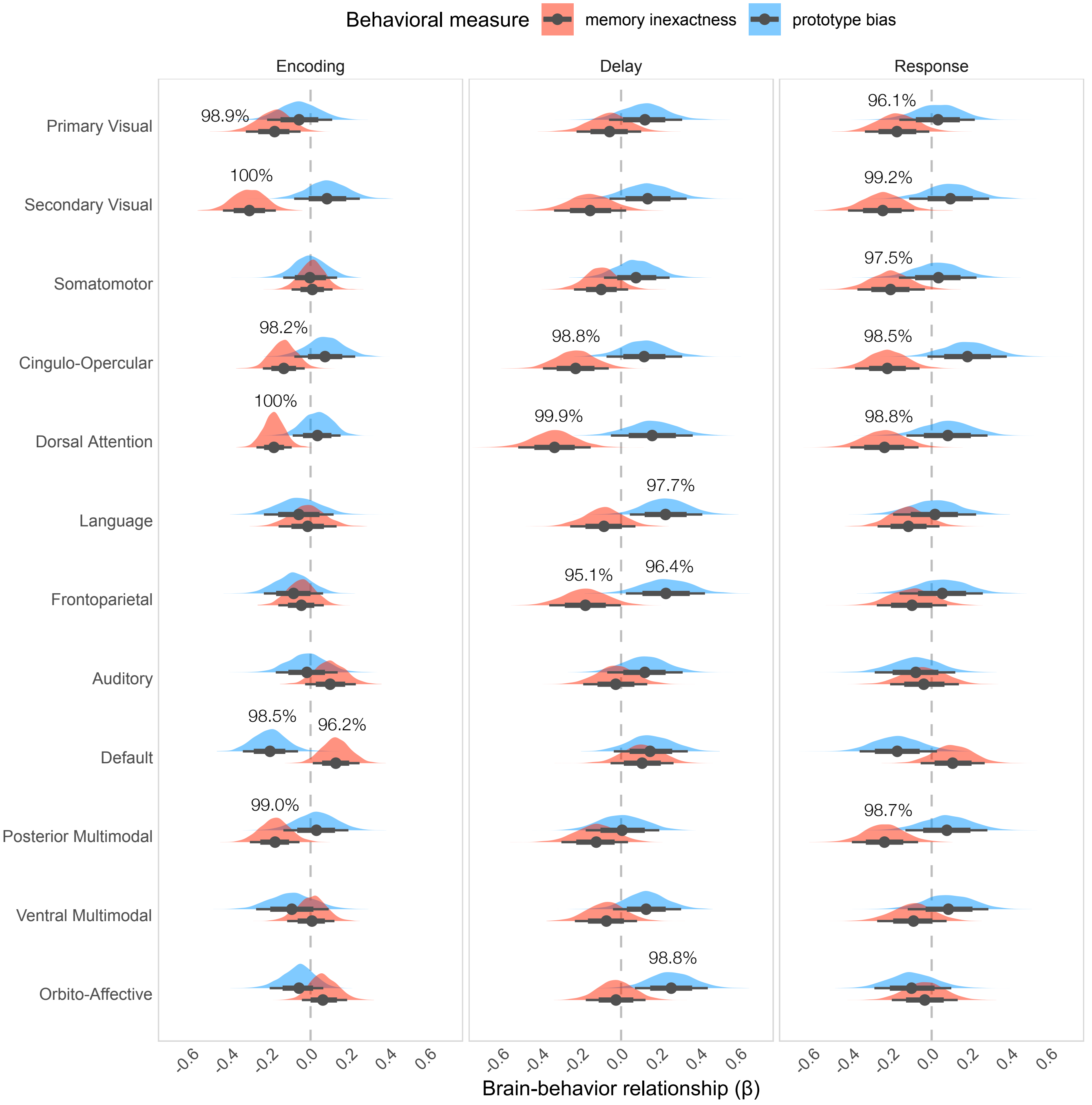

Figure S5. The relationship between the activity in different brain networks and individual spatial working memory performance. The relationship between brain activity and behavioral measures was estimated by running a Bayesian hierarchical linear model across participants with factors memory inexactness and prototype bias, and study as a random effect for each task phase separately. Shown are posterior distributions of the relationship between the activity of specific networks and behavioral measures of memory inexactness (red) and prototype bias (blue) for the encoding, delay, and response phases of a task trial. Points indicate mean β-estimates, and lines 95% confidence intervals.

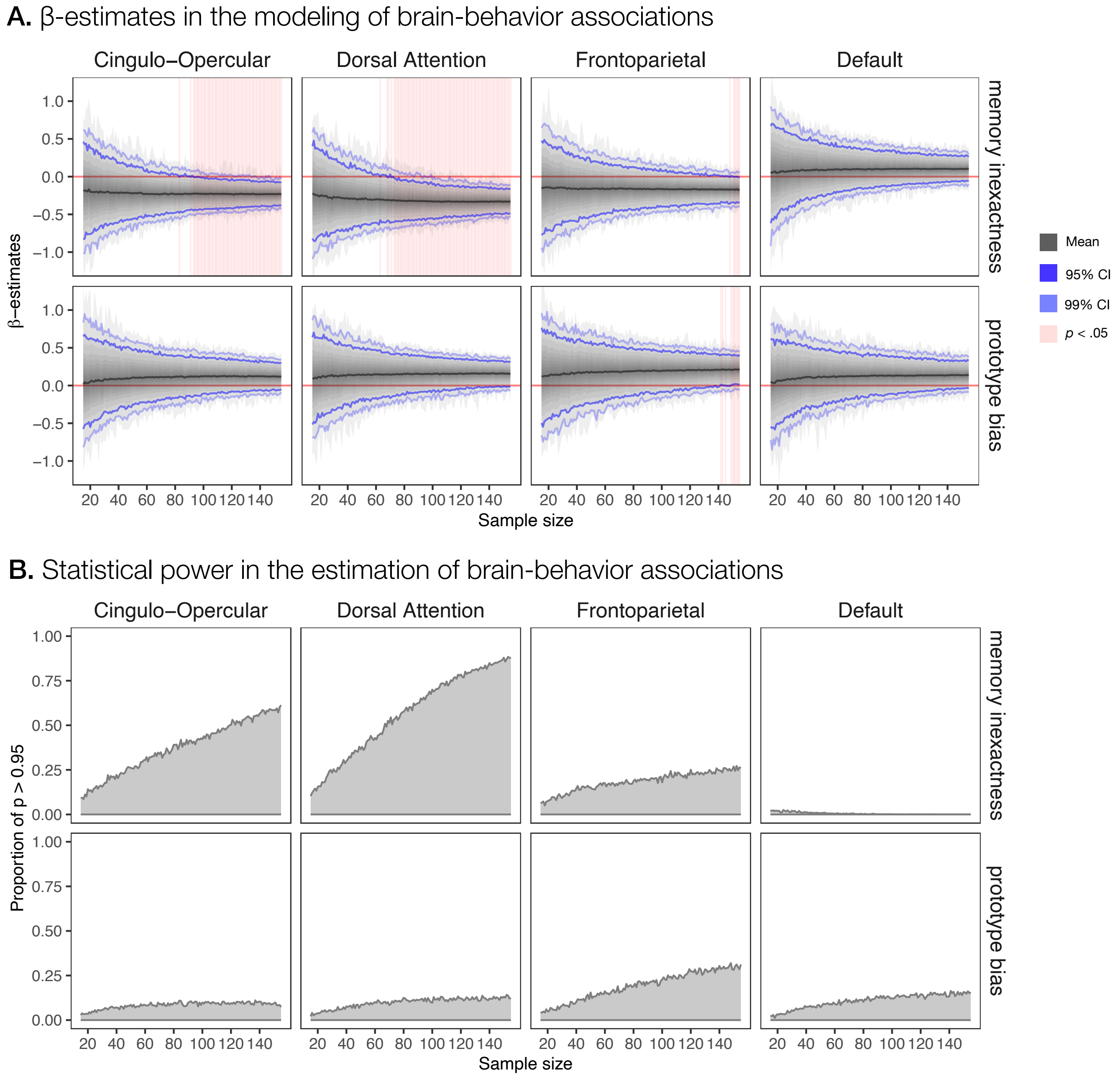

Figure S6. The effect of sample size on the estimation of brain-behavior relationships. We investigated the effect of sample size on **A.** β-estimates and **B.** statistical power in the investigation of the relationship of brain activity and behavioral measures of memory inexactness or prototype bias. The posterior probability of brain-behavior relationship was estimated using a Bayesian hierarchical linear model with factors memory inexactness and prototype bias, and study as a random effect, based on a varying number of sample sizes from 15 to 155. At each sample size, 1000 samples were created, each by sampling with replacements from the set of all participants. **A.** The black line denotes mean across all samples, the grayed area denotes the span between maximum and minimum value with the darker shading reflecting higher density, the lighter blue line denotes the upper and lower boundary for 99% of samples, the darker blue denotes the upper and lower boundary for 95% of samples. The red line denotes the value 0, the pink background shading denotes the sample sizes for which the 95% confidence interval does not include 0. **B.** The proportion of samples where 95% of posterior distribution was above or below 0.
